# Septic Shock and Spontaneous Gangrenous Gas Necrosis of the Spleen Secondary to *Clostridium perfringens*: The Importance of Source Control

**DOI:** 10.1155/2021/5563071

**Published:** 2021-05-06

**Authors:** Morgan Oskutis, Matthew Reaven

**Affiliations:** Division of Pulmonary, Allergy, Critical Care, and Sleep Medicine, Department of Medicine, Emory University School of Medicine, 615 Michael Street, Suite 205, Atlanta, GA 30322, USA

## Abstract

*Clostridium perfringens* is a rare cause of septic shock, occurring most frequently in immunocompromised patients. An uncommon cause of *Clostridium perfringen* septicemia is spontaneous gangrenous gas necrosis of the spleen, where the primary treatment is splenectomy. We present a case of septic shock caused by spontaneous gangrenous gas necrosis of the spleen secondary to *Clostridium perfringens* in a patient whose profound pancytopenia made obtaining definitive source control extremely difficult.

## 1. Introduction

*Clostridium perfringens* is a Gram-positive, rod-shaped, spore-forming, anaerobic bacterium [1]. It was first described in 1891 but became more recognized during War World One secondary to the prevalence of gas gangrene in soldiers [1]. Today, it is known to be pervasive throughout the environment being found in soil, sewage, various foods, and in the gastrointestinal tract of both healthy and unhealthy hosts [1]. Traumatic events that involve a breach of the body's integumentary with exposure to the environment are a well-established clinical scenario that places one at risk of developing a *Clostridium perfringen* infection [2]. Spontaneous gas gangrene, meaning infection that occurs without a preceding traumatic event, has been documented in the literature. It occurs almost exclusively in immunocompromised hosts and has a higher mortality than traumatic *Clostridium perfringen* infections [3].

Spontaneous gas gangrene of the spleen caused by *Clostridium perfringens* is a rare entity limited to a few case reports over the last 40 years [4–9]. The definitive treatment for such infection is splenectomy; although, percutaneous drainage has been described with success in non-*Clostridium perfringen* splenic abscesses [9, 10]. Here, we report a case of an immunocompromised female with gangrenous gas necrosis of the spleen in which source control was made difficult by pancytopenia.

## 2. Case Report

**Figure 1:**
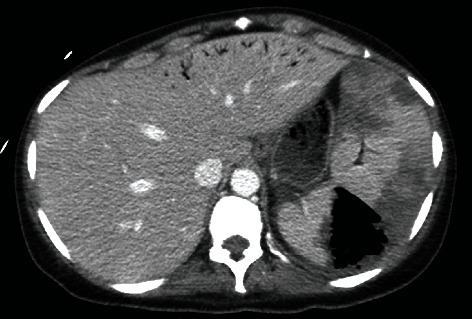
Axial CT image showing a markedly enlarged spleen (20 cm) with multiple areas of hypoattenuation indicating infarction, as well as a large wedge-shaped area of intrasplenic air.

A 50-year-old female with a history of myelofibrosis status post allogeneic peripheral blood stem cell transplantation complicated by graft-versus-host disease and hemolytic anemia secondary to major ABO incompatibility on prednisone, tacrolimus, and rituximab presented to our emergency department with complaints of shortness of breath and abdominal distension. Upon evaluation, the patient was found to be tachypneic, tachycardic, and requiring supplemental oxygen via nasal cannula. Abdominal examination was notable for mild left lower quadrant tenderness with no peritoneal signs. Laboratory evidence demonstrated pancytopenia (WBC 3.0 × 10^9^ cells/L, Hgb 6.2 g/dL, Plts < 2 × 10^9^ cells/L), elevated lactic acid, mildly elevated transaminases, and acute hyperbilirubinemia. The patient was transfused platelets and packed red blood cells in addition to crystalloid fluid. Blood cultures were obtained followed by the initiation of broad-spectrum antibiotics with ceftazidime and vancomycin. A computed tomography (CT) scan of the chest, abdomen and pelvis with intravenous contrast was obtained. The CT scan of the chest was notable for bilateral peripheral and basilar predominant ground glass and consolidative pulmonary opacities most concerning for atypical or viral pneumonia. The CT of the abdomen and pelvis (Figure 1) demonstrated an enlarged, 20 cm spleen with multiple wedge-shaped regions of hypoattenuation consistent with acute splenic infarcts. A dominant wedge-shaped area in the posterior superior spleen containing a significant amount of intrasplenic and subcapsular air was noted with continuation of the air tracking into the splenic vein and the portal venous system. These findings were most consistent with splenic necrosis. The patient was admitted to the medical intensive care unit for further management of septic shock.

Shortly upon arrival to the medical intensive care unit, the patient required intubation for increased work of breathing in the setting of septic shock. Infectious disease, acute care surgery, interventional radiology, hematology, and the bone marrow transplant services were all consulted to assist with management. Within less than 12 hours, the patient's blood cultures were positive for gram variable rods in all bottles. The patient's antibiotics were broadened to meropenem, vancomycin, and doxycycline. Given her underlying comorbidities, micafungin was also initiated out of concern for possible fungal infection. Acute care surgery and interventional radiology deemed the patient unsuitable for any immediate invasive intervention secondary to the patient's profound and refractory thrombocytopenia (platelet count < 2 × 10^9^ cells/L) and anemia (hemoglobin 5.8 g/dL) which did not improve despite multiple transfusions.

Over the next 24 hours, in hopes of preventing ongoing hemolytic anemia, the patient underwent her first out of five exchange transfusions which was performed without complication. At 48 hours after admission, the patient's initial blood cultures speciated to *Clostridium perfringens*. The respiratory viral panel, SARS-CoV-2 PCR, and the BIOFIRE® FILMARRAY® Pneumonia *plus* Panel were all negative. Vancomycin and doxycycline were discontinued while micafungin and meropenem were continued given high concern for additional enteric organism involvement.

Ninety-six hours after admission, the patient remained pancytopenic. Colony-stimulating growth factor was given in an attempt to improve her leukopenia. A repeat CT scan of the abdomen and pelvis demonstrated decreased splenic gas with resolution of portal venous and splenic vein gas. No discrete splenic abscess was identified. A transthoracic echocardiogram was negative for valvular vegetations. The second set of blood cultures obtained 48 hours after admission was no growth to date. The patient was hemodynamically stable without the need for vasopressors and was on room air after a self-extubation event.

Despite such medical treatment, the patient's third set of collected blood cultures for the hospitalization returned positive for *Clostridium perfringens* on hospital day six. Given high concern for lack of source control, a third CT abdomen and pelvis was performed which more clearly demonstrated a large splenic abscess. Given improvement in the patient's leukopenia, hemodynamics, and now available HLA matched platelets, interventional radiology and surgery were reengaged for possible intervention. On hospital day eight, the patient underwent successful percutaneous splenic drain placement. Intraoperative cultures collected from the spleen grew *Clostridium perfringens.*

The percutaneous splenic drain was removed on hospital day 23 after CT imaging demonstrated improvement, but not complete resolution of the splenic abscess. The patient's antibiotics were narrowed to ertapenem with an anticipated six-week course. The patient's remaining hospital course was complicated by acute encephalopathy, persistent pancytopenia requiring stem cell boost, and stercoral colitis. Ultimately, the patient was discharged to a rehabilitation facility on hospital day 40.

Twenty-seven days after discharge from the hospital and eleven days after the completion of her six-week course of ertapenem, the patient returned to the hospital febrile, tachycardic, and with a CT abdomen and pelvis demonstrating reaccumulation of splenic air and fluid. Blood cultures were again positive for *Clostridium perfringens.* Despite the risks given the patient's pancytopenia, the patient underwent preoperative splenic embolization with interventional radiology followed by a total splenectomy the following day by acute care surgery. The patient spent several days in the surgical intensive care unit but was ultimately discharged from the hospital on postoperative day 11. Pathology from the spleen demonstrated a 12.5 × 8.5 × 7.5 cm abscess cavity. Microbiology performed on the spleen grew *Clostridium perfringens.* Blood cultures collected after splenectomy remained negative for *Clostridium perfringens* suggesting appropriate source control.

## 3. Discussion

In this case report, we present an immunocompromised patient who developed *Clostridium perfringen* septicemia secondary to gangrenous gas necrosis of the spleen. Consistent with most cases reported in the literature, our patient was immunocompromised which made her susceptible to such infection. Although the exact etiology is unknown, we postulate that she experienced autoinfarction of her spleen from rapid growth which ultimately created an anaerobic environment where translocation of *Clostridium perfringens* from the gastrointestinal tract could migrate and proliferate. Such autoinfarction of the spleen in the setting of a hematological malignancy has been previously reported [11].

The major challenge in the management of this case was obtaining source control in the setting of profound and refractory thrombocytopenia and anemia. Decisions on platelet thresholds for various invasive procedures are mostly based on expert opinion rather than randomized control trials [12]. The Society of Interventional Radiology recommends a platelet count of greater than 50 × 10^9^ cells/L prior to performing a percutaneous splenic abscess drainage [13]. For major surgery, including splenectomy, the AABB also recommends a platelet count of greater than 50 × 10^9^ cells/L [14]. However, despite multiple platelet transfusions, exchange transfusions, and maximal medical management, this threshold was unable to be met. Given the less invasive nature, a decision was made to attempt percutaneous drainage with hopes of achieving source control with a lower risk of major bleeding. Unfortunately, despite drainage, the patient had persistent bacteremia. Therefore, splenectomy was ultimately performed despite the inability to achieve a platelet count above the recommended level.

This case is unusual in that to the authors' knowledge, there have been no cases described in the literature of a splenic abscess caused by *Clostridium perfringens* in which the patient's underlying comorbidities made immediate surgical intervention not possible. Although there is ample data demonstrating success of percutaneous drainage of splenic abscesses and even some reports of using medical therapy alone, none of these cases involve *Clostridium perfringens* [10, 15, 16]. In our case, percutaneous drainage plus antimicrobial therapy was insufficient in obtaining source control. It is possible that percutaneous splenic drainage and medical therapy are successful for certain bacteria and not others. This is an area for future research.

## 4. Conclusion

Spontaneous gangrenous gas necrosis of the spleen caused by *Clostridium perfringens* is a rare cause of septic shock. Management involves appropriate antimicrobial therapy combined with source control. Definitive source control is obtained by splenectomy. However, certain contraindications exist that may prohibit definitive source control. In this case, the patient's refractory pancytopenia made surgical intervention challenging. Attempts at percutaneous drainage were insufficient at obtaining source control resulting in persistent *Clostridium perfringen* bacteremia. High-risk splenectomy was ultimately performed which resulted in successful resolution of infection.

## Data Availability

No datasets were used.
